# Imaging of Cerebral Blood Flow in Patients with Severe Traumatic Brain Injury in the Neurointensive Care

**DOI:** 10.3389/fneur.2014.00114

**Published:** 2014-07-07

**Authors:** Elham Rostami, Henrik Engquist, Per Enblad

**Affiliations:** ^1^Section of Neurosurgery, Department of Neuroscience, Uppsala University, Uppsala, Sweden; ^2^Department of Neuroscience, Karolinska Institutet, Stockholm, Sweden; ^3^Department of Surgical Sciences, Anaesthesiology and Intensive Care, Uppsala University, Uppsala, Sweden

**Keywords:** cerebral blood flow, brain injury, neurointensive care, Xenon-CT, imaging

## Abstract

Ischemia is a common and deleterious secondary injury following traumatic brain injury (TBI). A great challenge for the treatment of TBI patients in the neurointensive care unit (NICU) is to detect early signs of ischemia in order to prevent further advancement and deterioration of the brain tissue. Today, several imaging techniques are available to monitor cerebral blood flow (CBF) in the injured brain such as positron emission tomography (PET), single-photon emission computed tomography, xenon computed tomography (Xenon-CT), perfusion-weighted magnetic resonance imaging (MRI), and CT perfusion scan. An ideal imaging technique would enable continuous non-invasive measurement of blood flow and metabolism across the whole brain. Unfortunately, no current imaging method meets all these criteria. These techniques offer snapshots of the CBF. MRI may also provide some information about the metabolic state of the brain. PET provides images with high resolution and quantitative measurements of CBF and metabolism; however, it is a complex and costly method limited to few TBI centers. All of these methods except mobile Xenon-CT require transfer of TBI patients to the radiological department. Mobile Xenon-CT emerges as a feasible technique to monitor CBF in the NICU, with lower risk of adverse effects. Promising results have been demonstrated with Xenon-CT in predicting outcome in TBI patients. This review covers available imaging methods used to monitor CBF in patients with severe TBI.

## Introduction

Traumatic brain injury (TBI) is one of the leading causes of death and disability in young adults. Acute TBI is characterized by a primary and a secondary injury. Primary brain injury is the direct injury to the brain parenchyma at the time of the initial impact, which can be both focal and diffuse depending on the biomechanics of the impact. The secondary brain injury is caused by a combination of neuronal and vascular damage, proteolytic pathways, excitotoxicity, oxygen-free radicals, apoptosis, inflammatory processes, and ischemia (1). Secondary brain injury may also be caused by secondary clinical events, e.g., increased intracranial pressure (ICP), hypotension, hypoxemia, seizures, and pyrexia (2).

Ischemia plays a major role in the pathology of TBI; signs of ischemic brain damage are found on autopsy in more than 90% of TBI patients (3, 4). Astrup et al. demonstrated that the critical ischemic cerebral blood flow (CBF) threshold for electrical/functional disturbance was higher than the threshold for complete electrical failure/infarction, which was the foundation for the ischemic penumbra concept (5, 6). Jones et al. showed that the risk of infarction was dependent both of the CBF level and duration of time with decreased CBF (7). Furthermore, the degree and duration of decreased CBF was shown to be decisive of the outcome (8) and correlated with injury severity following TBI (9, 10).

Another important observation was the “luxury perfusion syndrome” originally described by Lassen (11). This syndrome was described as cerebral hyperemia or “over-abundant CBF relative to the metabolic needs of the brain tissue.” Cerebral hyperemia following brain injury has been demonstrated in several studies using intravenous Xenon-133 (12, 13). Normal and high CBF with narrow arteriojugular venous difference of oxygen (AVDO_2_) indicating “luxury perfusion” has been shown in comatose TBI patients (14).

Areas surrounding the focal regions of reduced CBF display milder reductions in flow following TBI (15, 16). It has been suggested that this area is at risk for secondary insults but most importantly, due to its viability, it is sensitive to therapeutic interventions (17).

The brain has almost no capacity to survive without oxygen. Its reserved oxygen capacity will only last for a few seconds. The brain adenosine triphosphate (ATP), the fuel crucial for neuronal functioning, will only last for 40 s during complete ischemia (18). Furthermore, the level of ATP in brain tissue following TBI has been shown to correlate to the severity of the brain injury (19).

Mean arterial pressure (MAP), ICP, PaCO_2_, and PaO_2_ are the physiologic variables that would influence CBF. The most important relationship, however, is the principle of flow-metabolism coupling whereby cerebral metabolic rate of oxygen (CMRO_2_) is directly related to CBF and AVDO_2_ (20).

Changes in CBF following TBI in patients have been described as a triphasic pattern. During the first and acute phase, there is a 50% decrease in CBF (21, 22). During the following initial 12 h post-injury, the second phase begins, marked by a rise in CBF that approaches or exceeds normal values in some patients and typically persists for the next 4–5 days (23). This phase is followed in turn by a third period of low CBF that lasts for up to 2 weeks post-injury. However, there is a strong heterogeneity of CBF among TBI patients. In some patients, the CBF may persist low while in others normal CBF values can be seen that remains for days to weeks. The assessment of CBF following TBI is important to detect episodes of ischemia. Ischemia in TBI patients regardless of when detected has shown to be correlated with poor outcome (21, 23, 24). It is important to relate CBF changes to the metabolic demand where both metabolic depression and transient hyperglycolysis following TBI have been observed (25–27). The episodes of high CBF paralleled the episodes of hyperglycolysis and the question was raised whether low CBF in patients following TBI always means an inadequate supply for metabolic demand. Combining CBF imaging with microdialysis could show that high lactate/pyruvate ratio, usually interpreted as ischemia, occurred despite that the CBF was not low (28). This suggested that the metabolic crises can be caused by mitochondrial dysfunction rather than inadequate CBF.

A great challenge for the treatment of TBI patients in the neurointensive care unit (NICU) is to detect early signs of secondary brain injury in order to prevent further advancement and deterioration of the brain tissue. Multimodality monitoring using focal and global methods provides valuable information but has its limitations. Imaging of the brain may add important information. An ideal imaging technique would enable continuous non-invasive measurement of regional blood flow and metabolism across the whole brain. Currently, there are several methods that offer snapshots of the CBF, and some also provides information about the metabolic state of the injured brain. The aim of this article is to give a short review of currently available brain imaging methods used in patients with severe TBI. Relevant references are summarized in Table 1.

**Table 1:** **Presents a summary of publications using Xenon-CT, PET, perfusion CT, perfusion-MRI, and comparative studies using different imaging modalities**.

Reference	Subject	Additional monitoring	Time post-injury	*n*	GCS
**XENON-CT**					
Yonas et al. (29, 30)	CBF and TBI			1	GCS ≤8
Langfitt et al. (31)	Imaging pathology and neuropsychological tests (133Xe)	MRI, PET, CT, Xenon-133	Acute and 6 months	3	GCS ≤13
Latchaw et al. (32)	CBF in different types of TBI		During NIC stay	35	GCS ≤8
Marion et al. (21)	Xenon-CT and ICP		Within 7 days	23	GCS ≤8
Schalen et al. (33)	CBF and hyperventilation response (133Xe)	Jugbulb	During NIC stay	38	GCS ≤8
Marion et al. (21)	CBF following TBI		1 h–7 days	32	GCS ≤7
Bouma et al. (23)	Early measurement of CBF in TBI	Jugbulb	First 24 h	186	GCS ≤8
Bouma et al. (22)	CBF in acute phase of severe TBI		3 h	35	GCS ≤8
Bouma et al. (22)	Autoregulation test (133Xe)		First days	47	GCS ≤8
Stringer et al. (34)	CBF and hyperventilation		During NIC stay	12	
Bouma and Muizelaar (35)	Xenon-CT evaluation in TBI				
McLaughlin and Marion (36)	CBF and vasoresponsivity in contusions		24–48 h	10	GCS ≤8
Kelly et al. (37)	CBF and outcome (133Xe)		1–5 days	54	GCS ≤12
Schroder et al. (38)	Relation of CBV and CBF to explain cause of ischemia	Dynamic CT imaging (CBV)	24 h	51	GCS ≤8
Bouma et al. (39)	Underlying cause of cerebral swelling	Dynamic CT imaging (CBV)	Early posttrauma	37	
Doppenberg et al. (40)	Relation of potassium, glutamate, lactate, and CBF	Microdialysis		70	GCS ≤8
Kushi et al. (41)	CBF, C02, pH and CMR02 as prognostic indicator	Jugbulb		22	GCS ≤8
Hoelper et al. (42)	rCBF and contusions		29 and 94 h	44	GCS ≤8
von Oettingen et al. (43)	TBI and pulmonary trauma, xenon concentration and CBF			5	
Schutt et al. (44)	Thermo-dye-dilution and Xenon-CT	Thermo-dye-dilution	1–5 days	16	GCS ≤8
Valadka et al. (45)	CBF and pbr02	pbr02		18	GCS ≤6
Furuya et al. (46)	CBF and hypodense area			50	GCS ≤8
Chieregato et al. (47)	rCBF in traumatic hemorrhagic contusions			14	GCS ≤9
Chieregato et al. (48)	CBF and hypertension			7	GCS ≤8
Chieregato et al. (49)	Recovery of CBF in tICH		First 20 day	22	GCS ≤8
Inoue et al. (50)	CBF and 6 months outcome		1,2, 3, 4, 6 weeks	20	GCS ≤8
Chieregato et al. (51)	CBF in hematomas and traumatic contusions			43	GCS ≤8
Poon et al. (52)	C02 reactivity and cerebral hemodynamics	TCD, ICP, Microdialysis		35	GCS ≤12
Marmarou et al. (53)	Type of edema in diffuse and focal TBI	DWI, MR		45	GCS ≤8
Chieregato et al. (54)	CBF and CPP levels	Jugbulb	During NIC stay	237	GCS ≤8
Masaoka (55)	CBF during hypothermia	Jugbulb	1–4 days	30	GCS ≤8
Robertson et al. (56)	Genetic polymorphism of N03 and CBF		12 and 48 h	51	GCS ≤8
Kaloostian et al. (57)	Outcome prediction within 12 h following TBI		12 h	120	GCS ≤8
Shafer et al. (58)	CBF and oxygen saturation	INVOS	During NIC stay	22	“Intubated”
**PET**					
Bergsneider et al. (27)	Cerebral hyperglycolysis in TBI patients		1–24 h	28	GCS ≤8
Hattori et al. (59)	Metabolic rate of glucose and level of consciousness		1–5 days	23	GCS ≤15
Coles et al. (60)	Measurement of ischemic lesion		1–24 h	12	GCS ≤12
Coles et al. (61)	Ischemic brain volume and outcome		1–72 h	15	GCS ≤12
Hattori et al. (62)	characterize contusional, pericontusional, and remote regions in TBI		1–5 days	21	GCS 3-15
Wu et al. (63)	Metabolic rate of glucose in gray matter		1–4 days	14	GCS ≤14
Cunningham et al. (64)	Threshold for irreversible tissue damage		1–5 days	14	GCS ≤8
Vespa et al. (28)	Microdialysis and PET		Mean 36 h	19	GCS ≤14
**SPECT**					
Abdel-Dayem et al. (65)	SPECT correlated to CT		24 h	14	GCS ≤8
Roper et al. (66)	SPECT correlated to CT		72 h	15	GCS ≤15
Ito et al. (67)	Cerebral perfusion in diffuse brain injury and relationship to atrophy		Within 1 week and 1–6 months	8	GCS ≤8
**PERFUSION CT**					
Wintermark et al. (68)	Correlation of CPP and CBF			61	GCS ≤8
Wintermark et al. (69)	Prognostic value of admission CBF		At admission	130	GCS ≤10
Soustiel et al. (70)	Prognostic value of admission CBF		At admission and l week	30	GCS ≤9
Huang et al. (71)	Progression of cerebral contusions		1–6 h	22	GCS ≤8
Bendinelli et al. (72)	CBF within the first 48 h following TBI		1–48 h	30	GCS ≤8
**PERFUSION-MRI**					
Garnett et al. (73)	rCVB in contusion		2–19 days	18	GCS ≤8 vs. ≥9
**COMPARATIV STUDIES**					
Hagen et al. (74)	Comparison PW-MRI and Xenon-CT		1–24 h	10	Stenosis, HCF, atrophy
Rempp et al. (75)	PW-MRI and PET in healthy subjects			12	NC
Gillard et al. (76)	Comparison CTP and PET in AVM and gliomas			8	8 AVM, 2 glioma
Campbell et al. (77)	Comparison PW-MRI and perfusion CT in ischemic stroke		3–6 h	49	Acute stroke

*CBF, cerebral blood flow; ICP, intracranial pressure; CPP, cerebral perfusion pressure; CT, computer tomography; rCBV, regional cerebral blood flow; tICH, traumatic intracranial hematoma; pbrO2, partial brain oxygen tension; Jugbulb, jugular bulb; INVOS, brain tissue oxygen saturation*.

## Positron Emission Tomography

Positron emission tomography (PET) is an imaging technique that provides quantitative measurement of cerebral perfusion and metabolism (78, 79). Positron emitting radionuclides are used either after incorporation into chemical compounds (e.g., C^15^O, C^15^O_2_, and H_2_^15^0) or as molecular tracers (e.g., ^15^O_2_). After intravenous administration or inhalation, the isotopic tracer is distributed in the tissues according to the physiological properties, and the radioactive tracer concentration is measured regionally. The radioisotopes, which are produced by a cyclotron, have short half-life and must be used without delay. The detected regional tracer concentrations allows for mathematical quantification of CBF, cerebral blood volume (CBV), the oxygen extraction fraction (OEF), and CMRO_2_ (80–82).

O^15^-radioisotope has been used to measure CBF and OEF, while F^19^ deoxyglucose (FDG) assesses regional brain glucose metabolism. FDG is analogous to glucose in the body. It rapidly crosses the blood–brain barrier and is taken up into brain cells. Since FDG is not further metabolized, it can be imaged to produce metabolic maps for PET imaging. The images obtained are then generally co-registered with CT or magnetic resonance imaging (MRI) to obtain anatomic relationships (78, 79, 83).

Several studies have used PET in assessment of TBI (27, 28, 59, 60, 62–64, 84–86).

Regional brain ischemia was detected in TBI patients, who underwent PET within 24 h following injury according to CBF, OEF, and CMRO_2_ measurements, and an increase of the ischemic volume was correlated with poor outcome (61).

By using PET, both hypo- and hyperglycolysis have been demonstrated in TBI patients. In a study performed by Bergsneider et al., it was shown that both global and regional hyperglycolysis occur following severe TBI. Within 1 week following injury, 56% of the patients showed evidence of hyperglycolysis using FDG-PET (27). In a follow-up study, PET was performed within 4 days following TBI, and a depressed cerebral metabolic rate of glucose was observed (63).

Combining PET with microdialysis in TBI patients, Vespa et al. demonstrated high lactate/pyruvate ratio that indicates metabolic crises without presence of ischemia according to PET (28). It was suggested that high lactate/pyruvate ratio under those circumstances indicated mitochondrial dysfunction rather than ischemia (28, 87, 88).

One of the advantages of PET is that it can generate images of greater resolution. The main advantage is that PET provides the possibility to obtain quantitative regional measurements of both CBF and metabolism. The drawbacks are that the generation of isotopes requires a costly cyclotron and is labor intensive. In addition, the isotopes that are used have a shorter half-life thereby requiring that the radiopharmaceuticals must be readily available. Furthermore, PET provides a non-continuous measurement and the need for transfer of the patient to a radiology unit (61, 89).

### Single-photon emission computed tomography

The principal of single-photon emission computed tomography (SPECT) is similar to PET in using radioactive material to detect gamma rays. The tracer usually used is the radioisotope ^99m^Tc that crosses the blood–brain barrier and localize in the brain tissue in proportion to blood flow. SPECT is a semi-quantitative CBF measurement technique that is based on the calculation of tracer uptake ratios and an estimation of the relative regional CBF (rCBF) distribution within the brain (90).

An initial study lesions detected by SPECT in TBI patients during the first 72 h was compared to CT findings. It was shown that SPECT detected 40% more lesions than shown by CT scans (65). Similar results have been reported by Roper et al. (66). In a study of eight patients with severe diffuse TBI, SPECT revealed several lesions in all patients, even in non-depicted regions on MRI (67). Abnormalities in cerebral perfusion were also observed 1–6 months following injury.

There are numerous publications proving SPECT functional brain imaging as a powerful research tool, especially in the fields of cerebrovascular disease and cognitive disorders, and there is extensive development in receptor and molecular imaging (91). In a recent systematic review, Raji et al. concluded that SPECT imaging could be useful in evaluating patients with neurological and psychiatrically sequela after TBI (92).

Although SPECT provide images with lower resolution than PET, the radioisotope is significantly less expensive and longer lasting with no need of cyclotrons. Implementation of SPECT into clinical routine for estimation of rCBF in the acute phase of TBI is however lacking.

## Stable Xenon Computed Tomography

Xenon computed tomography (Xenon-CT) utilizes inhalation of a gas mixture containing 28% (30–35% in older studies) non-radioactive xenon (^131^Xe) and oxygen. The Xenon gas is a radio opaque, highly lipid soluble and capable of crossing the BBB. The Kety–Schmidt equation is applied to measure regional and global CBF. It provides primarily measurements of rCBF in the cortex and has been used under a variety of clinical conditions to study the pathophysiology and guide therapy regarding blood pressure and ventilation management (29). The first report of Xenon-CT in a TBI patient demonstrated that this technique could be used to detect regions with loss of autoregulation and CO_2_ reactivity (30). In a following Xenon-CT study including 35 TBI patients, the feasibility of using Xenon-CT for detecting changes in CBF, for guidance of the degree of hyperventilation, and for the identification of brain death was evaluated (32). Higher incidence of ischemia (very low CBF) than previously assumed following TBI could be demonstrated by Xenon-CT (35), which also was shown to be associated to high early mortality (22). Studies on contusions following TBI have shown that the CBF is concentrically distributed, improving from the core to the periphery (47). In traumatic hematomas, it was shown that low rCBF levels seen in the perihematoma low-density area CBF recovered from day 2 and forward and persistence ischemia was only seen in the core (49). It appears that type of contusion is also determinant of rCBF, with lower values in mixed contusions compared to hamorrhagic and non-hamorrhagic contusions (42).

The Xenon-CT has also been used to evaluate the effect of different interventions performed in the NICU on CBF following TBI. Chieregato et al. observed that rCBF in the intracontusional low-density area having critically reduced initial values was marginally affected by norepinephrine induced increase in cerebral perfusion pressure (CPP). However, in subjects starting from non-critical baseline values, rCBF was significantly reduced (48). Stringer et al. reported that hyperventilation in patients with acute brain lesion reduced CBF substantially both within the lesion and in apparently normal brain regions (34). Furthermore, the CBF in contusions has been shown to be reduced but with preserved CO_2_ reactivity. It was suggested that this area and the surrounding parenchyma are hypersensitive to hyperventilation and hypotension and thus vulnerable to secondary injury (36). The CO_2_ reactivity has been suggested as a good early prognostic indicator (41). In the study by Kushi et al., jugular bulb catheters were used in combination with Xenon-CT. Xenon-CT with simultaneous multimodality monitoring have generated interesting results regarding CBF and metabolism following TBI. Combining Xenon-CT with cerebral microdialysis showed that loss of CO_2_ reactivity was associated with increased ICP and increased lactate, glutamate and glycerol dialyzate levels, and consistently a fatal outcome (52). In another study, areas with low CBF showed increased levels of glutamate (40).

Xenon computed tomography has also been combined with tissue oxygen tension monitoring in TBI, and it showed a linear relationship between pbrO_2_ and CBF (45).

Promising results have been demonstrated with Xenon-CT in predicting outcome in TBI patients. Patients with low CBF that returns to normal 2–3 weeks following severe TBI show better neurological outcome that the group who had sustained low CBF (50).

In a recent study, Xenon-CT was used to monitor CBF in the first 12 h following injury in 120 TBI patients. It was shown that measurement of CBF within the first 12 h predicts 6 months outcome, and the best outcome prediction was obtained if Xenon-CT was measured within 6 h following TBI (57). In TBI, patient with pulmonary injury care should be taken in CBF analyses since end-tidal and arterial xenon concentration curves can be a source of error (43).

Previous studies indicate that endothelial nitric oxide synthase (NO_3_) activity is important in maintaining CBF after TBI. Using Xenon-CT, Robertson et al. studies the association of genetic polymorphism of NO_3_ and CBF following TBI. They showed that patients with the C/C genotype had lowest CBF values and worse outcome (56).

Xenon computed tomography is fast and provides quantitative measurements of CBF. The big advantage is that by using a mobile CT scanner there are no need to transport the patient to a different setting, which is always associated with increase risk of adverse events (93, 94). Other advantages are the short half-life and very low risk of side effects of inhaled Xenon, which makes repeated examinations more feasible. The very low risk of side effects was demonstrated in a recent multicentre evaluation study concluding that CBF measurements by Xenon-CT can be performed safely, with a very low risk of adverse events, and no risk of permanent morbidity or sequela could be detected (95). However, the brain is exposed to a small dose of radiation.

Wintermark et al. reported a radiation dose of 3.5–10 mSv for the whole xenon study (96). While Seifert et al. report that in the case of brain partially located in the region of primary radiation, a mean organ dose of 39 mSv was calculated, corresponding to an effective does of 1.6 mSv (97).

In our hands, the effective dose for a complete Xenon-CT examination is 2.7 mSv (in-house investigation, data not published). This dose can be compared to an annual mean background dose in Sweden of 3 mSv.

## Brain Perfusion Imaging

Cerebral perfusion is defined as the steady-state delivery of blood to brain tissue per unit volume, measured in milliliters per 100 g of tissue per minute. Perfusion imaging uses intravascular tracers most commonly iodine or gadolinium contrasts depending on imaging method. These methods take advantage of signal changes that accompany the passage of tracer through the cerebrovascular system. Contrasts used in MRI, such as gadolinium, are compounds that work by altering the magnetic properties of nearby hydrogen nuclei, while CT perfusion uses iodine contrast agents, which are radiopaque (such as iohexol) (98, 99).

The data acquired can be used to generate color-coded maps and quantification of various perfusion parameters, including CBF, CBV, mean transit time (MTT), and time to peak (TTP; the time from the start of contrast agent injection to the time of maximum enhancement). The two commonly used methods are perfusion-MRI and CT perfusion and will be discussed below.

### Perfusion-weighted MRI

The most common technique for measuring perfusion parameters with MRI is dynamic susceptibility contrast (DSC). DSC-MRI perfusion is also known as bolus-tracking MRI or perfusion-weighted MRI (PW-MRI). We chose PW-MRI throughout this review. In PW-MRI, a time series of fast T2-weighted images are acquired when gadolinium contrast agent is injected. As gadolinium passes through the tissues, it produces a reduction of T2 intensity depending on the local concentration. Curves showing intensity changes based on the concentration of gadolinium over time can be generated. This time-to-signal intensity alteration can be processed to extract parameters that reflect either CBF, CBV, or MTT. These are linked by the equation CBF = CBV/MTT, also known as the “central volume principle” (100, 101).

Studies on TBI using PW-MRI are limited. Garnett et al. used PWI during the subacute phase of TBI in a small group of patients and reported that regions of both normal-appearing and contused brain may have an abnormal regional CBV and had worse outcome (73). In a study including 10 patients with neurological illness, PW-MRI was compared with Xenon-CT and showed a good correlation of rCBF between these methods (74).

Perfusion-weighted MRI can be used together with diffusion-weighted MRI (DWI), then the ischemic penumbra can be identified (102, 103). It can assess vascular patency if magnetic resonance angiography (MRA) is added (104). PW-MRI was used in healthy subjects to measure absolute values of rCBF and rCBV and compares these to values obtained by PET. The values were in agreement with previous findings using PET both in white and gray matter (75). In a recent study, PW-MRI was compared with CT perfusion, and it was found that PW-MRI is superior in precision and accuracy in identifying ischemic core and salvageable tissue (77).

One of the advantages of PW-MRI is that the contrast agent used is gadolinium that has fewer contraindications than iodinated contrasts.

However, compared to CT scan the PW-MRI is relatively time-consuming and more expensive. MRI scans are also less available and more difficult to monitor patients in.

### Computed tomography perfusion scan

In computed tomography perfusion (CTP), a native (unenhanced) CT is obtained followed by infusion of iodinated contrast. The concurrent acquisition of images after contrast injection allow for the measurement of changes in tissue attenuation that occur in the brain with time. Post processing of the data allows the generation of color-coded maps and quantification of various perfusion parameters, including CBF, CBV, MTT, and TTP. CTP has been mainly used in the management of patients with acute stroke and other cerebrovascular disorders since it provides an easy and practical way of obtaining both structural and CBF pictures. The first study performed with CTP in severe TBI included 130 patients and suggested that the number of arterial territories with low rCBV values was an independent prognostic factor regarding functional outcome (69). Same group examined the correlation of CPP and CTP findings. They could discriminate between two groups of patients; one without relationship between CBF and corresponding CPP values, considered as preserved autoregulation and the other group with strong correlation between CBF and CPP, interpreted as impaired autoregulation. Interestingly, the group with impaired autoregulation showed better clinical outcome (68).

Two studies have examined cerebral contusions using CTP in patients with TBI. In the first study, both contusional core and the surrounding brain tissue were examined. It was shown that early CBV and CBF maps corresponds better than an early native CT scan with the findings of non-contrast CT scans after 1 week, and therefore, CTP provides better early evaluation of tissue viability (70). In the second study, CTP was performed within 6 h of arrival, and contrast extravasation was predictive of hemorrhage progression (71). A recent study performed CTP during the first 48 h following severe TBI and confirmed abnormal perfusion following TBI and detected areas of hypoperfusion in 60% of patients that were not seen in conventional CT (72).

Measurement of CBF using CTP was compared with PET in patients with arteriovenous malformations and gliomas. Good correlation was found, although CTP had a tendency to overestimate CBF (76).

The advantages of CTP are that all units equipped with a CT scanner can obtain perfusion images and it is fast. Disadvantages include the exposure to radiation, potential for an allergic reaction to the iodine contrast, contraindication to use iodine contrast in case of renal insufficiency and the risk of inducing renal failure with repeated examinations, and the need to predetermine the regions of interest before the scan. Furthermore, although quantitative values can be acquired with perfusion CT, the accuracy of the flow values obtained has not been fully validated. It has not been determined if the references for normal CBF and critical CBF thresholds as measured with PET or Xenon-CT can be applied in perfusion CT. Perfusion CT uses an intravascular tracer to measure intravascular CBF, which likely reflects a different physiologic mechanism than that of PET and Xenon-CT, which employ a diffusible trace.

## Conclusion

Cerebral hypoperfusion and other CBF disturbances are common after TBI and play an important role in the development of secondary brain injury. It has been difficult to integrate methods for quantitative rCBF measurements in neurointensive care, which relies mainly on uni-focal or global monitoring methods to detect cerebral ischemia or to evaluate different treatments aimed at increasing CBF. There is a need of a rapid assessment of CBF in the NIC unit without transportation of the patient. Currently, bedside Xenon-CT is the only method fulfilling these criteria. All other methods discussed above require transfer of TBI patients to the radiological department. However, Xenon-CT can only provide quantitative measurements of CBF. In order to assess parameters such as CBV, CMR_glu_, and CMRO_2_, it is necessary to use perfusion imaging and PET. PET is a complex and costly procedure with a need of cyclotron, which has limited its use to few TBI centers. None of the current available methods provide continuous measurements of CBF.

Bedside Xenon-CT emerges as an economical and more accessible imaging technique with few adverse effects that can be used in the routine NIC to measure CBF following TBI.

## Conflict of Interest Statement

The authors declare that the research was conducted in the absence of any commercial or financial relationships that could be construed as a potential conflict of interest.
